# Supplementary material for: The causal role of circulating inflammatory markers in osteoporosis: a bidirectional Mendelian randomized study
Source: Front Immunol. 2024 Jul 18;15:1412298. doi: 10.3389/fimmu.2024.1412298 (PMC11291241; doi:10.3389/fimmu.2024.1412298)
Supplement: Supplementary file 1 [file DataSheet_1.zip › supplementary material/OSTEOPOROSIS-CIM/GCST90274763.tsv.gz_leave_one_out.pdf]

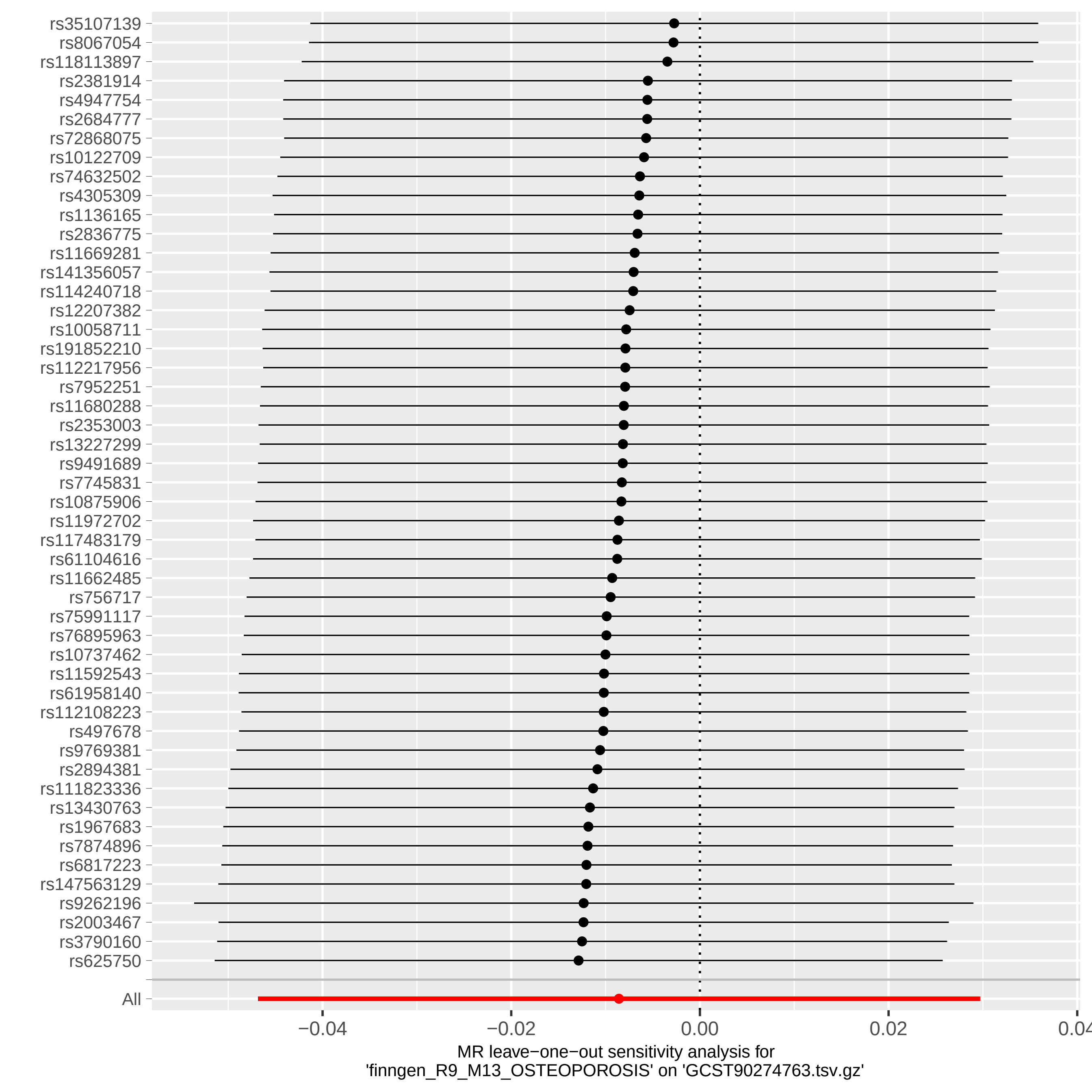
rs35107139
rs8067054
rs118113897
rs2381914
rs4947754
rs2684777
rs72868075
rs10122709
rs74632502
rs4305309
rs1136165
rs2836775
rs11669281
rs141356057
rs114240718
rs12207382
rs10058711
rs191852210
rs112217956
rs7952251
rs11680288
rs2353003
rs13227299
rs9491689
rs7745831
rs10875906
rs11972702
rs117483179
rs61104616
rs11662485
rs756717
rs75991117
rs76895963
rs10737462
rs11592543
rs61958140
rs112108223
rs497678
rs9769381
rs2894381
rs111823336
rs13430763
rs1967683
rs7874896
rs6817223
rs147563129
rs9262196
rs2003467
rs3790160
rs625750
All
−0.04
−0.02
0.00
0.02
0.04
MR leave−one−out sensitivity analysis for
'finngen_R9_M13_OSTEOPOROSIS' on 'GCST90274763.tsv.gz'
